# Excitatory and inhibitory neuron imbalance in the intrauterine growth restricted fetal guinea pig brain: Relevance to the developmental origins of schizophrenia and autism

**DOI:** 10.1002/dneu.22907

**Published:** 2022-11-24

**Authors:** Angela Cumberland, Nadia Hale, Aminath Azhan, Courtney P. Gilchrist, Ginevra Chincarini, Mary Tolcos

**Affiliations:** ^1^ School of Health and Biomedical Sciences RMIT University Bundoora Victoria Australia; ^2^ The Ritchie Centre, Hudson Institute of Medical Research Monash University Melbourne Victoria Australia; ^3^ Victorian Infant Brain Studies Murdoch Children's Research Institute Parkville Victoria Australia

**Keywords:** cerebral cortex, chronic placental insufficiency, cortical dysfunction, fetal growth restriction, hippocampus, interneuron, neuropsychiatric disorder

## Abstract

Neurodevelopmental disorders such as schizophrenia and autism are thought to involve an imbalance of excitatory and inhibitory signaling in the brain. Intrauterine growth restriction (IUGR) is a risk factor for these disorders, with IUGR onset occurring during critical periods of neurodevelopment. The aim of this study was to determine the impact of IUGR on excitatory and inhibitory neurons of the fetal neocortex and hippocampus. Fetal brains (*n* = 2) were first collected from an unoperated pregnant guinea pig at mid‐gestation (32 days of gestation [dg]; term ∼67 dg) to visualize excitatory (Ctip2) and inhibitory (calretinin [CR] and somatostatin [SST]) neurons via immunohistochemistry. Chronic placental insufficiency (CPI) was then induced via radial artery ablation at 30 dg in another cohort of pregnant guinea pigs (*n* = 8) to generate IUGR fetuses (52 dg; *n* = 8); control fetuses (52 dg; *n* = 7) were from sham surgeries with no radial artery ablation. At 32 dg, Ctip2‐ and CR‐immunoreactive (IR) cells had populated the cerebral cortex, whereas SST‐IR cells had not, suggesting these neurons were yet to complete migration. At 52 dg, in IUGR versus control fetuses, there was a reduction in SST‐IR cell density in the cerebral cortex (*p* = .0175) and hilus of the dentate gyrus (*p* = .0035) but not the striatum (*p* > .05). There was no difference between groups in the density of Ctip2‐IR (cortex) or CR‐IR (cortex, hippocampus) neurons (*p* > 0.05). Thus, we propose that an imbalance in inhibitory (SST‐IR) and excitatory (Ctip2‐IR) neurons in the IUGR fetal guinea pig brain could lead to excitatory/inhibitory dysfunction commonly seen in neurodevelopmental disorders such as autism and schizophrenia.

## 1 INTRODUCTION

Intrauterine growth restriction (IUGR) is the second leading cause of perinatal morbidity and mortality, exceeded only by prematurity (Gabbe & Annas, 1996). It is characterized by reduced growth velocity of the fetus, resulting in a failure of the fetus to attain its full growth potential (Thureen, Anderson, & Hay, 2001). IUGR can have a number of causes but is most commonly due to placental insufficiency via idiopathic impaired placentation (Baschat, 2004), which leads to chronic fetal hypoxemia, slowing of fetal growth, and compromise of several organ systems, including the developing brain. Brain regions of high vulnerability include the hippocampus and neocortex, as these regions develop over a protracted period, from fetal life through to the postnatal period.

Despite increased monitoring if growth restriction is identified in utero, and intervention to deliver the fetus prematurely if placental insufficiency is severe, children born following IUGR are predisposed to long‐term physical, cognitive, and mental health problems that are heterogeneous in presentation and severity. Deficits can range from physical impairments, behavioral and learning difficulties, and developmental and mental health disorders such as depression, schizophrenia, and autism spectrum disorders (ASD) (Abel et al., 2010; Cannon, Jones, & Murray, 2002; Ergaz, Avgil, & Ornoy, 2005; Geva et al., 2009; Huber, Ford, Bartlett, & Nathanielsz, 2015; Hultman, Sparén, & Cnattingius, 2002; Lampi et al., 2012; Ley, Laurin, Bjerre, & Marsal, 1996; Pallotto & Kilbride, 2006; Tideman, Maršál, & Ley, 2007; Zubrick et al., 2000). Indeed, structural changes in the brains of patients with schizophrenia and ASD are similar to those reported in IUGR children, suggesting there may be a similar etiology in the development of these disorders (Guilmatre et al., 2009; Owen, O'donovan, Thapar, & Craddock, 2011). It has recently been proposed that disorders such as schizophrenia and ASD arise from an imbalance of excitatory and inhibitory signals within the brain due to changes in populations of glutamatergic (excitatory) projection neurons and subpopulations of GABAergic (inhibitory) interneurons within the neocortex and hippocampus (Gao & Penzes, 2015; Gogolla et al., 2009; Kehrer, Maziashvili, Dugladze, & Gloveli, 2008; Yizhar et al., 2011). However, whether changes to these neuronal populations occur due to development in a compromised intrauterine environment as seen following IUGR is unknown.

Animal models in which placental insufficiency is induced provide an approach to investigate excitatory and inhibitory neuronal populations following IUGR. IUGR leads to a reduced density of glutamic acid decarboxylase 67 (GAD67)‐, calbindin‐, and calretinin (CR)‐immunoreactive (IR) neurons within the primary somatosensory cortex, but an increased density of GAD67‐ and CR‐IR neurons in the prefrontal cortex of adult rats (Delcour et al., 2012). In this previous study (Delcour et al., 2012), placental insufficiency was induced via unilateral uterine artery ligation at embryonic day (E) 17, just prior to term (E22) in pregnant rats, and thus represents a model of acute placental insufficiency. Here, we aim to determine whether IUGR induced via chronic placental insufficiency (CPI) in the pregnant guinea pig, a long gestation species with an intrauterine neurodevelopmental timeline similar to humans (see review by Morrison et al., 2018), affects early originating, COUP‐TF interacting protein 2 (Ctip2)‐IR subcortical excitatory projection neurons, and two subclasses of GABAergic inhibitory interneurons—somatostatin (SST)‐IR and CR‐IR.

## 2 MATERIALS AND METHODS

Experiments were approved by the University of Melbourne Animal Experimentation and Ethics Committee and were carried out in accordance with the National Health and Medical Research Council of Australia and international guidelines.

### 2.1 Surgery

CPI was induced in date‐mated Dunkin–Hartley guinea pigs (*n* = 4) at 30 days of gestation (dg; term ∼67 dg), using the diathermic ablation method as previously published (Kelleher, Palliser, Walker, & Hirst, 2011; McKendry, Palliser, Yates, Walker, & Hirst, 2010; Turner & Trudinger, 2009). Pregnant dams were anesthetized (ketamine 40 mg/kg [Illium Laboratories, Victoria, Australia] and xylazine 6 mg/kg [Troy Laboratories, NSW, Australia, intramuscular]) and a midline incision was made in the abdominal wall exposing the peritoneal cavity. The uterine horns were exteriorized exposing the uterine vessels and every second radial artery leading to an individual fetus’ placenta was cauterized. Sham (control) surgery (*n* = 4) was performed the same way but without cauterization of vessels. The muscle wall and skin were sutured closed, and the area was swabbed with chlorhexidine/ethanol solution and a topical antibiotic powder (Cicatrin^®^ powder; GlaxoSmithKline, Middlesex, UK) was applied. All surgical procedures were performed under aseptic conditions. Guinea pigs were left to recover on a heat pad before being transferred to a large box for housing. An additional pregnant guinea pig did not undergo CPI or sham surgery and served as an absolute control to qualitatively assess the spatial and temporal development of Ctip2‐IR subcortical projection neurons, and SST‐ and CR‐IR interneurons around the time of CPI surgery (i.e., at 32 dg).

### 2.2 Animals

At 32 and 52 dg, pregnant guinea pigs and fetuses were deeply anesthetized (Lethabarb; sodium pentobarbitone, 130 mg/kg; intraperitoneal; Virbac Pty. Ltd., Australia), and fetuses were delivered via cesarean section. At 52 dg, fetuses and placentae were weighed and the crown–rump length (CRL) was measured prior to perfusion fixation and tissue collection; these data have previously been published (Tolcos et al., 2018). Fetal guinea pigs were considered to be growth restricted (IUGR) based on established and previously published criteria: (i) mean body weight and CRL were 2 standard deviations (SD) below the mean weight for age‐matched controls, and (ii) mean brain weight to body weight ratio was 2 SD above the mean ratio for age‐matched controls (Jones, Lafeber, & Roebuck, 1984).

### 2.3 Perfusion and tissue preparation

Fetal guinea pigs (32 dg: control, *n* = 2 and 52 dg: control, *n* = 7; IUGR, *n* = 8) were transcardially perfused with 0.9% saline and 4% paraformaldehyde (PFA) in 0.1 M phosphate buffer (pH 7.4), and the brains were removed from the skull. Whole brain weights were recorded (52 dg only) and brains from both ages were post‐fixed in 4% PFA for 4 h at 4°C; control and IUGR brain weights at 52 dg have previously been published (Tolcos et al., 2018). At 52 dg, the brain was hemisected and the left cerebral hemisphere was embedded in paraffin, and serially sectioned (8 μm) from the midline in the sagittal plane with every 10th section collected on Superfrost^®^ Plus slides (Menzel‐Glaser, Braunscheig, Germany) for immunohistochemistry. At 32 dg, the whole brain was embedded in paraffin and was serially sectioned (8 μm) along the coronal plane.

### 2.4 Immunohistochemistry

For each animal at each age, three sections (80 μm apart) containing cerebral cortex, striatum, and hippocampus were immunostained for each antibody. Immunoreactivity was performed to identify neuron‐specific protein NeuN (Neuronal nuclei; mature neurons), SST (GABAergic interneurons), CR (GABAergic interneurons), and Ctip2 (early originating, subcortical excitatory projection neurons) using techniques previously described (Tolcos et al., 2011). Ctip2 was chosen as a marker of excitatory projection neurons as these cells establish themselves early in cortical development (Chen et al., 2008). Somatostatin and calretinin were chosen as markers of early and late migrating interneurons in rodents (Miyoshi et al., 2010; Xu, Cobos, De La Cruz, Rubenstein, & Anderson, 2004). All primary and secondary antibodies, dilutions, and suppliers are summarized in Table 1. Negative controls were performed by replacing the primary antibody with phosphate buffered saline (pH 7.4); in these experiments, immunoreactivity failed to occur. For each antibody, sections from each experimental group were stained simultaneously to ensure conditions remain identical to not bias subsequent analysis.

**TABLE 1:** Immunohistochemistry: Primary and secondary antibodies

1° antibody (dilution)	Localizes	Catalogue number; Supplier	2° antibody (dilution)
Mouse anti‐NeuN (1:500)	Mature neurons	Clone A60; Chemicon, MA USA	Biotinylated anti‐mouse IgG (1:200)
Mouse anti‐somatostatin (1:500)	Interneurons	Donated from Dr AMJ. Buchan (Costa et. al. 1980)	Biotinylated anti‐mouse IgG (1:200)
Rabbit anti‐calretinin (1:250)	Interneurons	7699/3H; SWANT, Bellinzona, Switzerland	Biotinylated anti‐rabbit IgG (1:200)
Rat Anti‐Ctip2 (1:200)	Deep cortical neurons	Ab18465; Abcam	Biotinylated anti‐rat IgG (1:200)

### 2.5 Qualitative assessment at 32 dg

**FIGURE 1:**
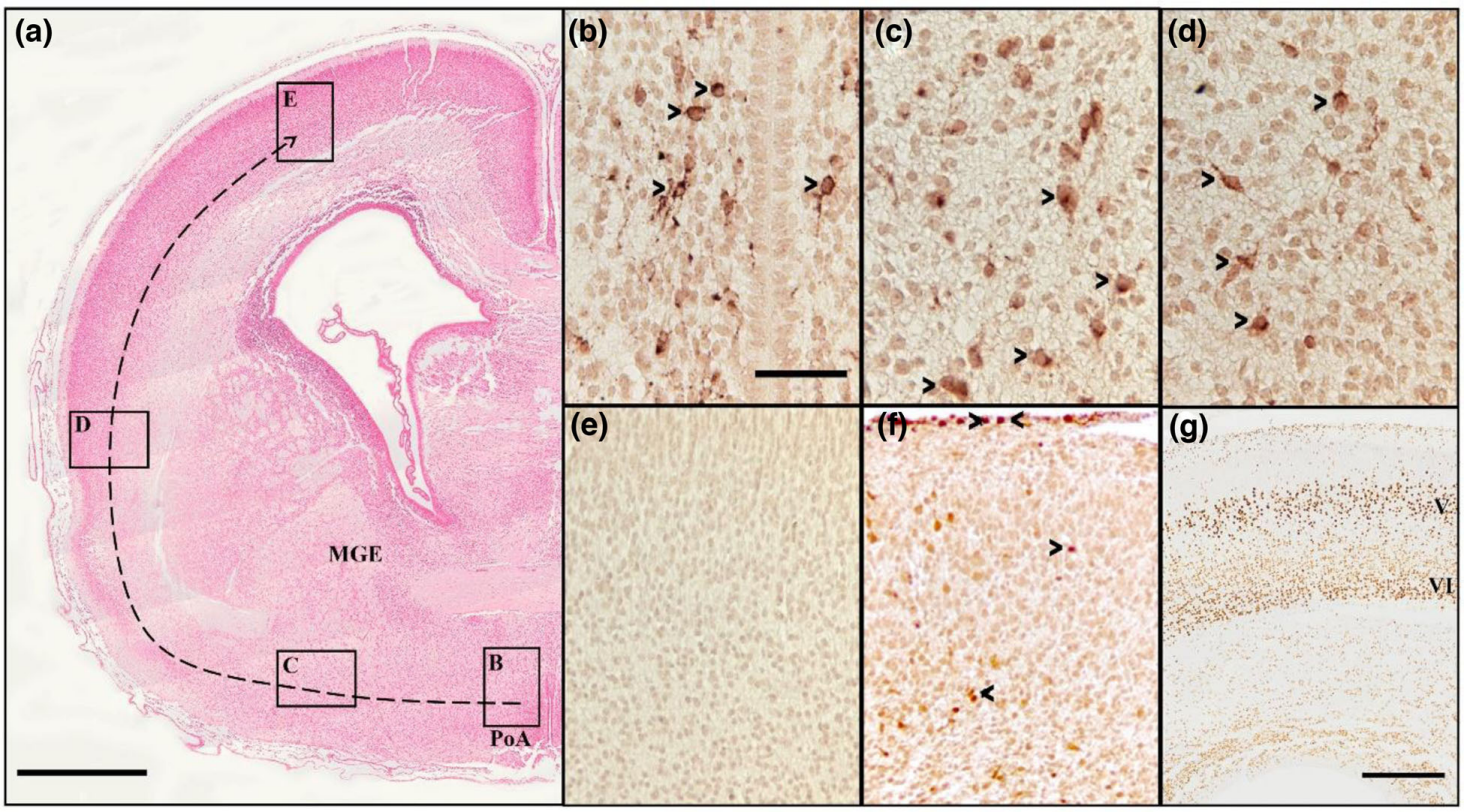
Distribution of SST‐, CR‐, and Ctip2‐immunoreactive (IR) neurons in the fetal guinea pig brain at 32 days of gestation (dg). (a) Photomicrograph of a right hemisection of a guinea pig brain at 32 dg stained with Eosin. Boxes in panel (a) correspond to images B–E and dotted arrow indicates the migratory pathway of SST‐IR neurons from the preoptic area (PoA) to the cerebral cortex. (b–d) At 32 dg in the guinea pig, SST‐IR neurons (arrowheads) were found in the PoA (b), en route to the cerebral cortex (c, d), but not in the cortex (e). (f) CR‐IR neurons (arrowheads) were present in the cerebral cortex (equivalent to location E). (g) Ctip2‐immunoreactivity in the cerebral cortex shows that layers V and VI, but not IV, are present in the fetal guinea pig brain at 32 dg. Scale bars: (a) 1 mm; (b–f) 200 μm; (g) 300 μm. MGE, medial ganglionic eminence

To assess the spatial and temporal development of SST‐, CR‐, and Ctip2‐IR neurons around the time of CPI induction, immunostaining was qualitatively assessed in coronal sections of fetuses (*n* = 2) from an unoperated dam at 32 dg. Sections were scanned using a Mirax Digital Slide scanner (Zeiss, Göttingen, Germany) and an image from four regions of interest (20× magnification) was taken starting from the preoptic area (PoA) and extending tangentially toward the cortical plate (Figure 1a).

### 2.6 Quantitative analysis at 52 dg

Immunostained sections were scanned using a Mirax Digital Slide scanner (Zeiss). Immunohistochemical analyses (e.g., cell density and area measurements) were performed using ImageJ software (http://fiji.sc/Fiji). Means were calculated for each animal and a mean of means for IUGR and control groups was determined; all densities are expressed as cells/mm^2^. Slides were coded by an independent researcher to prevent experimenter bias.

#### 2.6.1 Calculations of cerebral areas using NeuN

At 52 dg, digital images of NeuN‐immunostained sections from control and IUGR fetuses (*n* = 3 sections per animal) were traced using ImageJ software to obtain total cross‐sectional area of the cerebral hemisphere, cortical area (cerebral white matter [WM] + gray matter [GM]), cerebral WM and cerebral GM areas, cross‐sectional area of the total hippocampus (hippocampus proper + dentate gyrus [DG]), area of the hippocampus proper, and DG area (mm^2^). Ratios of the cortical area (GM + WM), cerebral GM area, and cerebral WM area to total cross‐sectional area of the cerebral hemisphere were also calculated.

#### 2.6.2 Areal density of SST‐, CR‐, and Ctip2‐IR neurons

**FIGURE 2:**
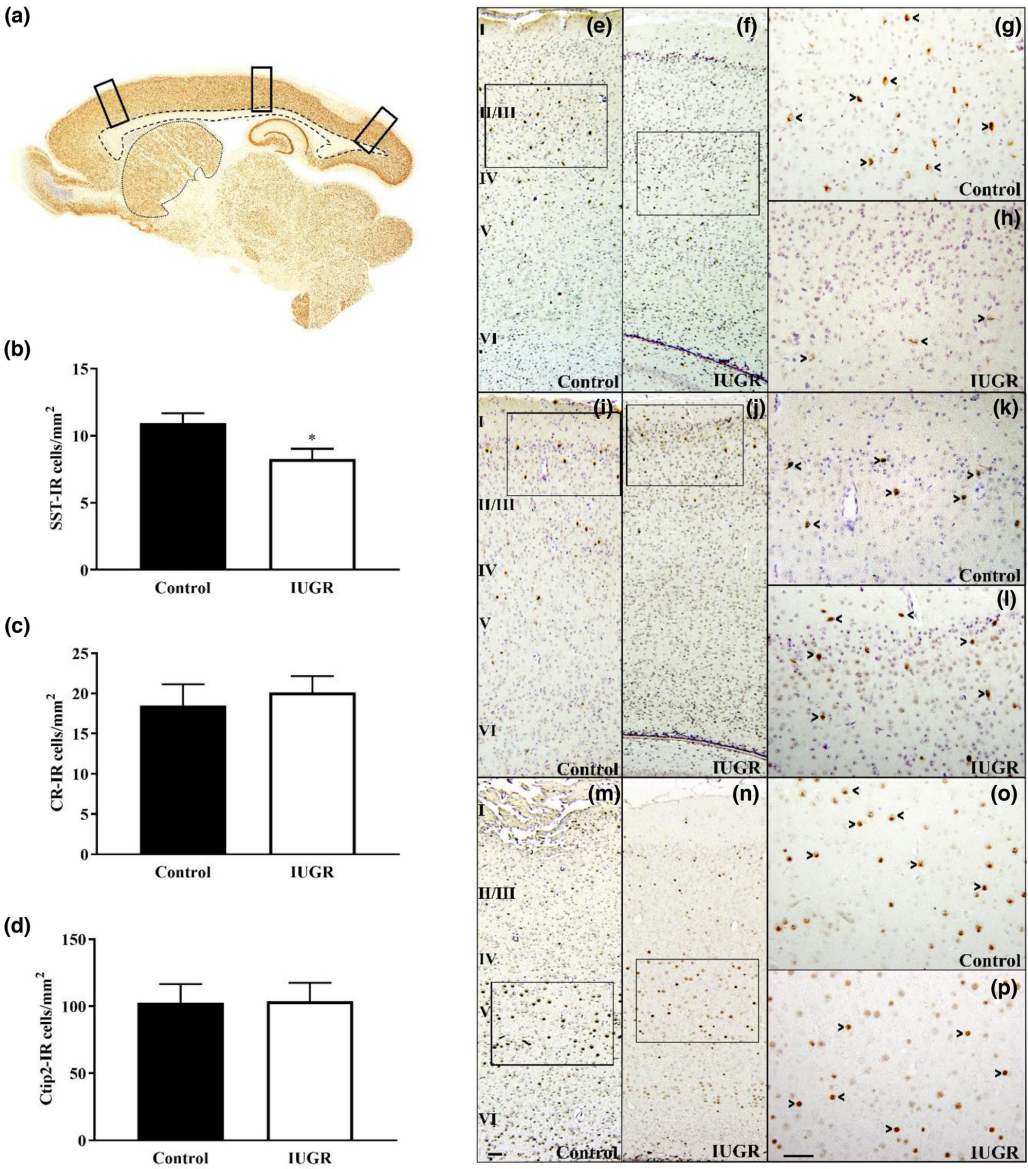
Analysis of SST‐, CR‐, and Ctip2‐IR neurons in cerebral cortex of control and IUGR guinea pigs at 52 dg. (a) NeuN‐immunostained sagittal section representing regions of interest extracted from the frontal, parietal, and occipital lobes (black columns); dashed line outlines the corpus callosum and dotted line outlines the striatum. (b) The areal density of SST‐IR cells was significantly reduced in the cerebral cortex in IUGR compared to control fetuses. However, there was no difference in the areal density of CR‐IR cells (c) or Ctip2‐IR cells (d) in the cortex of IUGR compared to control fetuses. (e–h) SST‐IR neurons were present throughout cortical layers II–VI in control (e, g) and IUGR (f, h) fetuses. (i–l) CR‐IR neurons were predominantly localized to cortical layers II and III in control (i, k) and IUGR (j, l) fetuses. (m–p) Ctip2‐IR neurons were localized to cortical layers V and VI in control (m, o) and IUGR (n, p) fetuses. Scale bars: (m) 100 μm; (p) 100 μm. Scale bar in panel (m) applies to panels (e), (f), (i), (j), (m), and (n). Scale bar in panel (p) applies to panels (g), (h), (k), (l), (o), and (p). Boxes in panels (e), (f), (i), (j), (m), and (n) are representative of locations of high‐power micrographs in panels (g), (h), (k), (l), (o), and (p), respectively. Arrowheads indicate immunopositive cells within each high‐power micrograph. Data are expressed as mean ± SEM. *p < .05. CR, calretinin; Ctip2, COUP‐TF interacting protein 2; I–VI, cortical layers 1–6; SST, somatostatin

At 52 dg, SST‐, CR‐, and Ctip2‐IR neurons were counted in three sagittal sections per animal, corresponding to lateral coordinates 1.90–2.10 mm of the rat atlas (Paxinos & Watson, 2009). Regions of interest were extracted from the frontal, parietal, and occipital levels of the cerebral cortex in columns that spanned the depth of cortical layers I–VI (400‐μm‐wide columns; 20× magnification; 3 columns/cortical region/animal). These columns were consistently taken from directly above the rostral, mid, and caudal aspects of the corpus callosum (Figure 2a), coinciding with the primary motor, primary somatosensory, and secondary visual cortices, respectively (Paxinos & Watson, 2009). The area of cerebral cortex present in these columns was measured and used to calculate areal density of SST‐, CR‐, and Ctip2‐IR neurons and expressed as cells/mm^2^. SST‐ and CR‐IR neurons were also counted in subregions of the hippocampus proper and DG (20× magnification) and in the entire cross‐sectional extent of the caudate putamen in three sagittal sections per animal (10× magnification). The area of these regions was measured and used to calculate areal density of SST‐ and CR‐IR neurons and expressed as cells/mm^2^.

### 2.7 Statistical analysis

Data at 52 dg were analyzed using GraphPad Prism Software (GraphPad Software Inc., La Jolla, CA, USA), with an alpha set at .05. Fetal physical parameters are expressed as mean ± SD; all other data are expressed as mean of mean ± standard error of the mean (SEM). Body morphometry and brain area data, and areal cell density of SST‐IR neurons in the striatum between control and IUGR offspring were assessed using independent samples *t*‐tests. A two‐way ANOVA was used to assess areal cell density of SST‐, CR‐, and Ctip2‐IR neurons with group (control and IUGR) and brain region (frontal, parietal, and occipital in the cortex; stratum oriens [SO] and stratum lacunosum moleculare [SLM] of the hippocampus proper; and the hilus and molecular layer (ML) of the DG) as main effects and a group‐by‐region interaction term. If a significant interaction was identified (interaction *p* < .05), Bonferroni post hoc testing was used to identify differences between control and IUGR groups in each brain region separately, and cell density is presented separately for each cortical region in all graphical representations. If there was no evidence that cell density differed across cortical regions (interaction *p* *>* .05), main effects for group are reported and group differences (control vs. IUGR) in cortical cell density are presented in graphical representations.

## 3 RESULTS

### 3.1 Body, brain, and organ weights at 52 dg

At 52 dg, there was a significant decrease in body weight (*t*_(13)_ = 3.13, *p* = .008), brain weight (*t*_(13)_ = 2.23, *p* = .044), liver weight (*t*_(13)_ = 2.54, *p* = .025), and placenta weight (*t*_(13)_ = 4.12, *p* = .001), and a reduction in CRL (*t*_(13)_ = 4.25, *p* = .001) in IUGR fetuses compared to controls (Table 2). There was an increase in brain to body weight ratio (*t*_(13)_ = 2.31, *p* = .038), and no difference in the brain to liver weight ratio (*t*_(13)_ = 1.67, *p* = .12) between IUGR and control fetuses (Table 2), indicating that IUGR fetuses displayed a degree of brain sparing, but were symmetrically growth restricted. Body weight, brain weight, CRL, and body to brain weight ratio have been previously reported (2018); the data are included in the present study to show that IUGR was achieved in this model.

**TABLE 2:** Body, brain, and organ weights of control and IUGR guinea pigs at 52 days of gestation

	Control (*n* = 7)	IUGR (*n* = 8)
Body weight (g)^a^	51.2 ± 6.42	40.16 ± 7.15**
Brain weight (g)^a^	2.02 ± 0.11	1.84 ± 0.19*
Liver weight (g)	3.09 ± 0.64	2.34 ± 0.52*
Placenta weight (g)	3.94 ± 0.39	3.22 ± 0.28**
CRL (cm)^a^	10.84 ± 0.24	9.41 ± 0.23**
Brain:body weight^a^	0.04 ± 0.001	0.05 ± 0.003*
Brain:liver weight	0.68 ± 0.14	0.82 ± 0.19

*Note*: Data are expressed as a mean ± SD.

Abbreviation: CRL, crown‐rump length.

^a^ Data previously published (Tolcos et al., 2018).

* *p* < .05

** *p* < .01.

### 3.2 Cerebral areas at 52 dg

In IUGR compared to control fetuses, there was a significant reduction in the total cross‐sectional area of the cerebral hemisphere (*t*_(11)_ = 3.01, *p* = .012; Table 3). There were no differences in cortical area (GM + WM; *t*_(11)_ = 0.57, *p* = .58) and cerebral GM area (*t*_(11)_ = 0.38, *p* = .71), but there was a trending reduction in cerebral WM area (*t*_(11)_ = 1.92, *p* = .081; Table 3). The ratio of cortical area to the total cross‐sectional area of the cerebral hemisphere (*t*_(11)_ = 3.83, *p* = .003) and the ratio of cerebral GM area to the total cross‐sectional area of the cerebral hemisphere (*t*_(11)_ = 3.22, *p* = .008) were significantly increased in IUGR compared to control fetuses (Table 3). There was no difference in the ratio of the cerebral WM to the total cross‐sectional area of the cerebral hemisphere (*t*_(11)_ = 0.96, *p* = .36; Table 3). In IUGR versus control, there were no differences in cross‐sectional area of the total hippocampus (*t*_(10)_ = 1.72, *p* = .12) or the DG (*t*_(10)_ = 0.92, *p* = .38), although there was a trending reduction in area of the IUGR hippocampus proper (CA subfields; *t*_(10)_ = 2.07, *p* = .065; Table 3).

**TABLE 3:** Morphological assessment of the cerebrum in control and IUGR fetuses at 52 days of gestation

	Control (*n* = 7)	IUGR (*n* = 8)
Total cross‐sectional area (mm^2^)	94.2 ± 4.1	80.8 ± 1.92*
Cortical area (GM + WM) (mm^2^)	40.2 ± 2.0	38.9 ± 1.2
Cerebral GM area (mm^2^)	31.8 ± 2.1	32.7 ± 1.4
Cerebral WM area (mm^2^)	8.5 ± 0.7	6.3 ± 0.9
Cortical area: total cross‐sectional area	0.43 ± 0.01	0.48 ± 0.01**
Cerebral GM: total cross‐sectional area	0.34 ± 0.02	0.40 ± 0.01**
Cerebral WM: total cross‐sectional area	0.09 ± 0.01	0.08 ± 0.01
Hippocampal area (hippocampus proper + DG) (mm^2^)	3.9 ± 0.3	3.3 ± 0.2
Hippocampus proper	3.1 ± 0.2	2.6 ± 0.1
DG	0.85 ± 0.1	0.72 ± 0.1

*Note*: Data are expressed as a mean ± SEM.

Abbreviations: DG, dentate gyrus; GM, gray matter; WM, white matter.

* *p* < .05

** *p* < .01.

### 3.3 SST‐, CR‐, and Ctip2‐IR neurons at 32 dg

At 32 dg, CR‐, SST‐, and Ctip2‐IR cells were present in the developing fetal guinea pig brain (Figure 1). SST‐IR cells were identified in the PoA, as well as along the tangential migratory path (Figure 1b–d); these neurons also had the morphology of migrating neurons with elongated and extended processes visible. SST‐IR neurons (Figure 1e) were not present in the cortex, but CR‐IR neurons were (Figure 1f). Ctip2‐immunostaining revealed that cortical layers V and VI had formed by 32 dg (Figure 1g).

### 3.4 SST‐, CR‐, and Ctip2‐IR neurons in the cerebral cortex at 52 dg

At 52 dg, SST‐IR neurons were distributed throughout cortical layers II–VI in control and IUGR fetuses (Figure 2b,e–h). There was no significant group‐by‐region interaction (*p* = .58) or main effect of region (*p* = .67) on SST‐IR cell density. There was a significant main effect of group (*F*_(1,37_ = 5.85, *p* = .021) denoting an overall reduction in cortical SST‐IR cell density in IUGR (8.23 ± 0.76 cells/mm^2^) compared to control fetuses (10.94 ± 0.74 cells/mm^2^; Figure 2e).

CR‐IR neurons were mainly in, but not restricted to, cortical layers I–III in control and IUGR fetuses (Figure 2i–l). There was no significant group‐by‐region interaction (*p* = .22) or main effect of group (*p* = .63) or region (*p* = .99) in the density of CR‐IR cells (Figure 2c).

Ctip2‐IR neurons were predominantly localized to layers V and VI, with some in layer IV, in control and IUGR fetuses (Figure 2m–p). There was no significant group‐by‐region interaction (*p* = .72) or main effect of group (*p* = .84) or region (*p* = .20) in the areal density of Ctip2‐IR cells (Figure 2d).

### 3.5 SST‐ and CR‐IR neurons in the hippocampus and striatum at 52 dg

**FIGURE 3:**
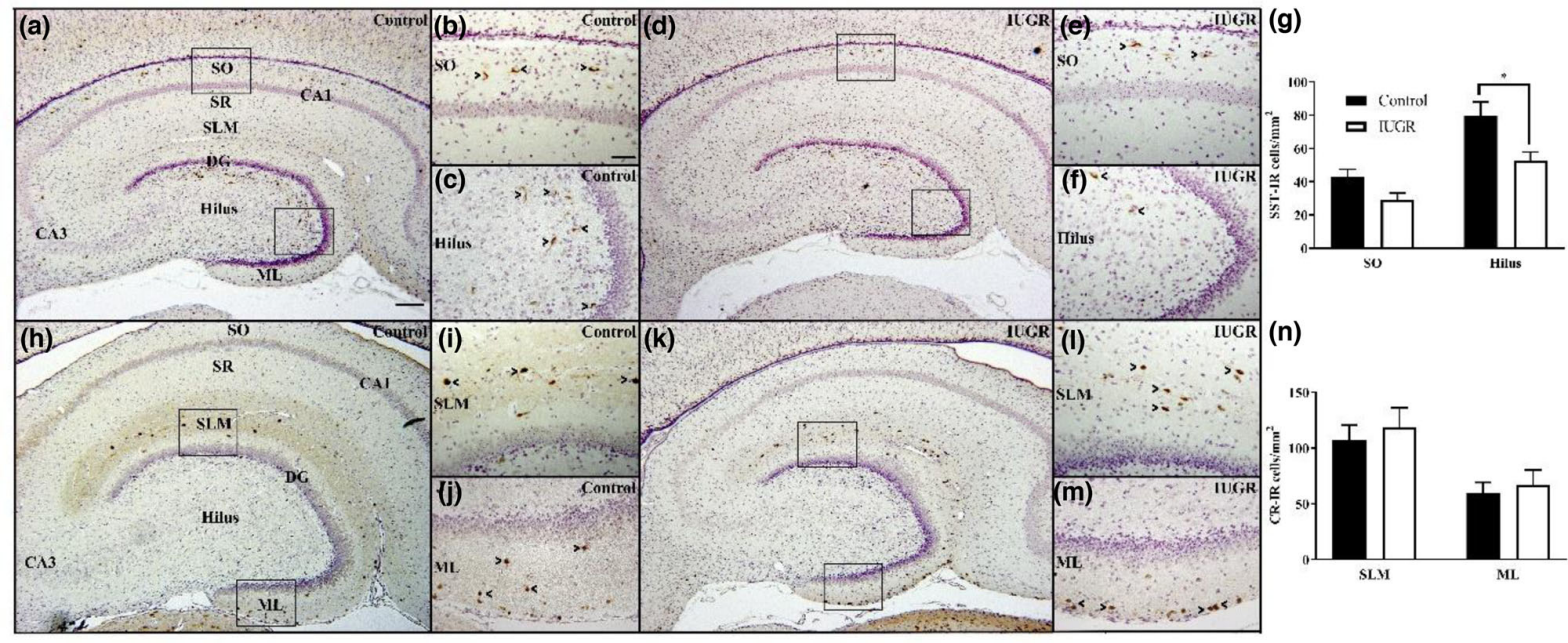
Analysis of SST‐ and CR‐IR neurons in hippocampus of control and IUGR guinea pigs at 52 days of gestation (dg). (a–f) SST‐IR neurons were localized to the SO of the CA1 region (b, e) and hilus region of the dentate gyrus (c, f) in control (a–c) and IUGR (d–f) fetuses. (g) There was a significant reduction in the areal density of SST‐IR neurons in the hilus region of IUGR (white bars; *n* = 8) compared to control (black bars; *n* = 7) fetuses. (h–m) CR‐IR neurons were localized to the SLM of the hippocampus (i, l) and ML of the dentate gyrus (j, m) in control (h–j) and IUGR (k–m) fetuses. (n) There was no difference in density of CR‐IR cells in either the SLM of the hippocampus proper or the ML of the DG in IUGR compared to control fetuses. Scale bars: (a) 300 μm; (b) 100 μm. Scale bar in panel (a) applies to panels (a), (d), (h), and (k). Scale bar in panel (b) applies to panels (b), (c), (e), (f), (i), (j), (l), and (m). Boxes in panel (a) represent location of high‐power images in panels (b) and (c); boxes in panel (d) represent location of high‐power images in panels (e) and (f); boxes in panel (h) represent location of high‐power images in panels (i) and (j); boxes in panel (k) represent location of high‐power images in panels (i) and (m). Arrowheads indicate positive cells within each high‐power micrograph. Data are expressed as mean ± SEM. *p < .05. CA1, Cornu Ammonis 1; CA3, Cornu Ammonis 3; CR, calretinin; DG, dentate gyrus; ML, molecular layer; SLM, stratum lacunosum moleculare; SO, stratum oriens; SR, stratum radiatum; SST, somatostatin

SST‐IR neurons were localized to the SO of the hippocampus proper and hilus region of the DG in IUGR and control fetuses (Figure 3a–f). There was no significant group‐by‐region interaction (*p* = .23). However, there was a significant main effect of group (*F*_(1, 26)_ = 13.67, *p* = .001) and region (*F*_(1, 26)_ = 30.04, *p* < .001). Post hoc analysis identified a significant reduction in the hilus region (control: 79.91 ± 8.04 cells/mm^2^; IUGR: 52.68 ± 5.12 cells/mm^2^; mean difference 27.22 [8.65, 45.8], *p* = .004) but not the SO (Figure 3g). There were also fewer SST‐IR cells/mm^2^ in the SO compared to the hilus in both control (SO: 42.83 ± 4.58 cells/mm^2^, hilus: 79.91 ± 8.04 cells/mm^2^; mean difference 37.07 [17.89, 56.26], *p* < .001) and IUGR fetuses (SO: 29.23 ± 3.91 cells/mm^2^, hilus: 52.68 ± 5.12 cells/mm^2^; mean difference 23.46 [5.51, 41.40], *p* = .009).

CR‐IR neurons were localized to the SLM of the hippocampus proper and ML of the DG in control and IUGR fetuses (Figure 3h–m). There was no significant group‐by‐region interaction (*p* = .88), nor a main group effect (*p* = .53) on the areal density of CR‐IR cells (*p* = .53) between IUGR (SLM: 118.54 ± 17.81 cells/mm^2^; ML: 66.90 ± 13.53 cells/mm^2^) and control fetuses (SLM: 107.02 ± 13.55 cells/mm^2^; ML: 59.89 ± 9.33 cells/mm^2^; Figure 3n). There was a significant main effect of region (*F*_(1, 25)_ = 11.72, *p* = .002), with the ML having fewer cells compared to the SLM (ML: 63.40 ± 3.50 cells/mm^2^; SLM: 112.78 ± 5.76 cells/mm^2^; mean difference 49.38 [19.68, 70.09]) irrespective of group (control and IUGR).

In the striatum, SST‐IR neurons were uniformly distributed throughout the caudate putamen, with no difference in areal density of SST‐IR (*p* = .79) between control (20.14 ± 1.33 cells/mm^2^) and IUGR fetuses (20.90 ± 2.32 cells/mm^2^). There were no CR‐IR cells in the striatum in control or IUGR fetuses and thus no further analyses were performed.

## 4 DISCUSSION

This study investigated the impact of IUGR on the prenatal development of specific subsets of excitatory (Ctip2‐IR) and inhibitory (SST‐IR and CR‐IR) neurons using a precocious species—the guinea pig. Our major findings were that in IUGR compared to control fetuses, there was a significant reduction in the areal density of SST‐IR cells throughout the cerebral cortex and the hilus region of the DG, but not in the SO of the hippocampus proper, or in the striatum. We also found no difference in the areal density of CR‐IR cells in the cerebral cortex and hippocampus, or Ctip2‐IR cells in the cerebral cortex in IUGR compared to control fetuses. Our data provide evidence that pregnancy compromises, such as IUGR, may contribute to an imbalance of excitatory and inhibitory neurons in the prenatal brain. This imbalance, primarily due to a reduction in a subset of inhibitory interneurons, may lead to cortical dysfunction and contribute to a predisposition for disorders such as ASD and schizophrenia in individuals born IUGR (Gao & Penzes, 2015; Selten, van Bokhoven, & Nadif Kasri, 2018).

### 4.1 SST‐IR but not CR‐IR inhibitory interneurons in the cerebral cortex are vulnerable to IUGR in the late gestation guinea pig

To date, no postmortem human or preclinical IUGR studies have investigated SST interneurons in the cerebral cortex. In the present study, we found an overall reduction in the areal density of SST‐IR cells in the cerebral cortex of the IUGR fetal guinea pig brain, but no evidence that these reductions differed across frontal, parietal, or occipital regions, implying that reductions in this inhibitory interneuron population were not region specific. Importantly, given the lack of difference between groups in cortical area, the decrease in areal density of SST‐IR cells in the IUGR fetal brain likely represents a reduction in total SST‐IR cell number. Given that SST interneurons are the second most abundant interneuron subtype in the neocortex (Lim, Mi, Llorca, & Marín, 2018), and account for approximately 30%–40% of the interneuron population in this region, a significant reduction in SST‐IR cells in the IUGR fetal brain may contribute to a loss in inhibitory cortical control via a dysregulation in modulating excitatory projection neurons. However, the neurological consequence and future clinical presentation of this cortical dysregulation in IUGR are unknown.

In contrast to the adverse effect of IUGR on SST‐IR inhibitory interneurons, the density of CR‐IR cells did not differ between control and IUGR late‐gestation fetuses. This finding suggests that CR‐IR interneurons are less vulnerable than SST‐IR interneurons to the impact of IUGR induced by CPI at mid‐gestation. This difference in interneuron population vulnerability may be explained by the timing of SST and CR interneuron development in the cerebral cortex of the fetal guinea pig. Indeed, we identified that in the mid‐gestation fetal guinea pig brain (i.e., at the time of CPI induction), SST‐IR interneurons are in the processes of migrating while CR‐IR interneurons have already migrated to their final cortical destination. This finding aligns with the timing of CR‐IR and SST‐IR interneuron migration in the fetal human brain (González‐Gómez & Meyer, 2014; Zecevic, Hu, & Jakovcevski, 2011) but contrasts that in mice and rats (Miyoshi et al., 2010; Xu et al., 2004), and highlights the importance of using neurobiological comparable species to model developmental brain injury.

### 4.2 Ctip2‐IR excitatory neurons in the cerebral cortex are not affected in the IUGR fetal guinea pig brain

In the present study, there was no difference in the density of Ctip2‐IR cells in the cerebral cortex of IUGR compared to control fetuses. As for CR‐IR interneurons, the lack of vulnerability of these excitatory subcortical projection neurons is likely due to the timing of CPI induction, as cortical layer V–VI Ctip2‐IR neurons were already present in the mid‐gestation fetal guinea pig brain. This suggests that cells (excitatory or inhibitory) in the process of migrating are more likely to be affected by CPI. Although CPI did not affect Ctip2‐IR excitatory projection neurons in the present study, the reduction in SST‐IR neurons could result in reduced inhibition of excitatory inputs, and thus lead to aberrant cortical excitation and impaired cortical function.

### 4.3 Differential impact of IUGR on hippocampal interneurons and hippocampal regions in the fetal guinea pig

Previous studies have reported no difference in the total population of GABAergic interneurons (i.e., GAD67‐positive) in the hippocampal CA1 region in CPI‐induced late‐onset IUGR fetal guinea pigs (Cumberland, Palliser, Rani, Walker, & Hirst, 2017) or in the DG in nutrient restriction‐induced IUGR postnatal rats (Ohishi et al., 2010). However, no studies have investigated specific hippocampal interneuron subpopulations in CPI‐induced IUGR. Here, we report that in IUGR compared to control fetuses the areal density of SST‐IR was reduced in the hilus of the DG, but there was no difference in the density of CR‐IR neurons in the SLM of the hippocampus proper or ML of the DG. Our findings in the hippocampus are in line with those for the cerebral cortex, that is, SST‐IR interneurons are more vulnerable to the impact of CPI‐induced IUGR in the fetal guinea pig brain. This finding is not surprising given that in rodents, SST‐IR and CR‐IR interneurons that populate the hippocampus originate and migrate from the same germinal regions as cortical interneurons (Fogarty et al., 2007; Pleasure et al., 2000). However, the vulnerability of SST‐IR interneurons to the impact of IUGR may also be region specific as we found no difference in the areal density of SST‐IR cells in the SO of the hippocampus. In the mid‐gestation fetal guinea pig brain, the CA1 region of the hippocampus has already begun to establish its distinct layers, while the DG is still in its primitive form (Mallard, Loeliger, Copolov, & Rees, 2000). Thus, the timing of IUGR onset in the present study aligns with a “window of vulnerability” of SST‐IR cells of hilus in the DG, more so than those of the SO of the hippocampus proper.

Both chronic hypoxia and inflammation are aspects of IUGR that could contribute to the cellular effects described in the cortex and hippocampus in this study. Studies investigating in utero hypoxic events using umbilical cord occlusion or carotid artery occlusion in fetal sheep report loss of interneurons, including parvalbumin‐ and SST‐positive interneurons in the cortex and hippocampus, respectively, over 1–2 weeks after hypoxic event (Ardalan et al., 2019; Fowke et al., 2018). Maternal immune activation by lipopolysaccharide (bacterial) and poly I:C (viral) also results in a loss of parvalbumin‐ and SST‐positive cells in multiple cortical regions, including motor, somatosensory, prelimbic, and anterior cingulate cortices in exposed fetal mice and rats (Lacaille et al., 2019; Vasistha et al., 2020). Therefore, chronic hypoxia and inflammation, alone or in combination, together with the timing of CPI induction, could be the driving factors for the reduction in SST‐positive interneurons in the cortex and DG identified in our study.

### 4.4 Relevance to schizophrenia and ASD

Impaired GABAergic neuronal function and signaling have been linked to neurodevelopmental disorders such as schizophrenia and ASD. At postmortem, individuals with schizophrenia have fewer small neurons (presumed to be GABAergic interneurons) in the prefrontal cortex, cingulate gyrus, and hippocampus (Benes, Kwok, Vincent, & Todtenkopf, 1998; Benes, McSparren, Bird, SanGiovanni, & Vincent, 1991), fewer reelin‐positive inhibitory cells and reduced GAD67 protein levels in the prefrontal cortex (Guidotti et al., 2000), and reduced SST mRNA expression in the prefrontal cortex and hippocampus compared to controls (Fung et al., 2010; Konradi et al., 2011; Morris, Hashimoto, & Lewis, 2008). In contrast, in the ASD brain, there is conflicting evidence for changes to GABAergic neurons. For example, GAD67 mRNA expression in Purkinje cells (Yip, Soghomonian, & Blatt, 2007) and GAD65 and GAD67 protein expression in cerebellar and parietal cortices (Fatemi et al., 2002) are reduced in the brains of ASD compared to controls at postmortem. Conversely, GAD67 mRNA is increased in inhibitory basket cells of the cerebellum (Yip, Soghomonian, & Blatt, 2008), and the density of parvalbumin‐, CR‐, and calbindin‐positive interneurons are increased in distinct regions of the hippocampus proper and DG in ASD brains (Lawrence, Kemper, Bauman, & Blatt, 2010). While there is still no consensus as to whether GABAergic neurons and/or signaling are increased or decreased in the ASD brain, and indeed which part of the brain is most affected, it can be agreed that the primary outcome is dysregulation in inhibitory control pathways. Whether this inhibitory dysregulation can also be attributed to an effect on SST‐expressing interneurons in ASD, as we have shown in the IUGR fetal guinea pig brain, has not yet been investigated.

Although the direct consequences of reduced GABAergic neurons and cortical inhibition in the human brain cannot be definitely known, use of rodent gene knockout models to reduce global GABAergic cell numbers has produced impaired social interactions and increased anxiety‐like behaviors (Levitt, 2005; Powell et al., 2003) reminiscent of altered social behaviors seen in schizophrenia and ASD. In GAD67‐deficient (as a model of schizophrenia) compared to wild‐type mice, males show greater avoidance in interacting with unfamiliar males, reduced preference for investigating unknown females over unknown males, a lack of social odor preference (Sandhu et al., 2014), and no preference between choosing an unfamiliar mouse over an object and a familiar mouse (Zhang, Hill, Labak, Blatt, & Soghomonian, 2014). Similarly, selective viral shRNA‐mediated SST knockdown in the prefrontal cortex and hippocampus of the adult rat (to mimic the pathological finding of decreased interneuron function in schizophrenia) results in an increased firing rate of dopaminergic neurons concurrent with reduced social interactions with an unfamiliar rat; this did not occur in parvalbumin knockdown rats (Perez, Boley, & Lodge, 2019), suggesting that a loss of SST‐expressing interneurons may be key in the development of schizophrenia‐like social deficits.

Specific ASD gene knockout models have also been used to study the relationship between interneuron subpopulations and behavioral outcomes. In *PTEN*‐knockout compared to wild‐type mice, the areal density of cortical SST‐IR interneurons is reduced (Vogt, Cho, Lee, Sohal, & Rubenstein, 2015), sociability and motor learning is impaired, and anxiety‐like behaviors are increased (Shin, Santi, & Huang, 2021). The loss of total SST‐IR cells in the DG of *Engrailed2‐*knockout compared to wild‐type mice (Provenzano et al., 2020; Sgadò et al., 2013) is associated with fewer reciprocal social behaviors, lack of preference toward investigating novel objects during testing, impaired prepulse inhibition (a measure of sensorimotor gaiting often impaired in these neurodevelopmental disorders), and poorer motor learning on the rotarod (Brielmaier et al., 2012). Taken together these studies highlight the impact of GABAergic neuron loss or dysfunction in the adult brain on social and anxiety‐like behaviors typically observed in ASD and schizophrenia. However, whether a reduction in GABAergic neurons or a discrete subset of these (e.g., SST‐expressing interneurons) in the developing brain leads to such long‐term behavioral changes is not known.

Individuals born IUGR have an increased risk of developing schizophrenia or ASD later in life (Abel et al., 2010; Lampi et al., 2012; Sacchi et al., 2020); however, the neurobiological link between IUGR and these neurodevelopmental disorders is not yet understood. Given that SST‐expressing interneurons are critical for maintaining excitatory/inhibitory balance in the brain and may be associated with the presentation of schizophrenia and ASD, deficits in SST‐expressing interneurons within the developing fetal brain may be an early risk factor for the development of these disorders. Thus, the reduction in SST‐IR cell density due to IUGR seen in the present study may contribute to an impaired inhibitory cortical network, which in part may contribute to the known predisposition of these infants to schizophrenia and ASD later in life, where aberrant excitation is a key factor.

### 4.5 Limitations of the study

The present study has examined the impact of IUGR on SST‐IR and CR‐IR inhibitory neurons and Ctip2‐IR pyramidal (excitatory) neurons the in fetal guinea pig brain. We propose that a reduction in SST‐IR, with no change to Ctip2‐IR cells, in the IUGR fetal cerebral cortex may result in cortical dysfunction, and a predisposition to neurodevelopmental disorders such as schizophrenia and ASD. Given our study was in the fetal guinea pig brain, we cannot comment on whether the alterations were sustained throughout postnatal development. Furthermore, although long‐term behavioral deficits have been reported in preclinical IUGR models (Batalle et al., 2014; Camprubí Camprubí et al., 2017; Delcour et al., 2012; Illa et al., 2013; Reid et al., 2012), including reduced prepulse inhibition in 12‐week‐old IUGR guinea pigs (Rehn et al., 2004), whether these behavioral changes are caused by, or associated with, a loss of SST‐IR neurons in the IUGR fetal brain is unknown. In addition, analysis of the impact of IUGR on other interneuron (e.g., parvalbumin, vasoactive intestinal polypeptide, reelin) and cortical pyramidal cell (e.g., Cux1, SatB2, NECAB1) populations may provide further evidence to help support the proposal that an excitatory/inhibitory imbalance in the fetal IUGR brain contributes to behavioral deficits that manifest later in life.

## 5 CONCLUSION

Using a model of CPI‐induced IUGR in the fetal guinea pig, we have shown that IUGR results in a reduction in the density of SST‐IR interneurons in the cerebral cortex and hilus of the DG, but not the hippocampal SO or the striatum, without impacting CR‐IR interneurons or Ctip2‐IR excitatory pyramidal neurons; these data highlight that there is a developmental window of vulnerability for different neuronal subtypes relative to the timing of the IUGR insult. Given that interneurons are critical for the proper development and function of the brain, a loss of inhibitory neuromodulation may potentially lead to aberrant excitation and excitatory/inhibitory imbalance.

This excitatory/inhibitory imbalance is a key theory in the development of neurological disorders such as autism and schizophrenia. This work highlights IUGR as a potential risk factor for the onset of the excitatory/inhibitory imbalance via the loss of SST‐expressing interneurons.

## AUTHOR CONTRIBUTIONS

All authors had full access to all the data in the study and take responsibility for the integrity of the data and the accuracy of the data analysis. Mary Tolcos conceptualized the idea of the study, performed supervision, and acquired funding. Mary Tolcos, Nadia Hale, and Aminath Azhan designed the methodology. Angela Cumberland, Nadia Hale, and Aminath Azhan performed investigation. Angela Cumberland and Nadia Hale performed formal analysis and wrote the original draft. Angela Cumberland, Nadia Hale, and Mary Tolcos provided resources. All authors reviewed and edited the manuscript. Angela Cumberland and Mary Tolcos performed visualization.

## CONFLICT OF INTEREST

The authors declare no conflict of interest.

## Data Availability

The data that support the findings of this study are available from the corresponding author upon reasonable request.
